# An extended patch-dynamic framework for food chains in fragmented landscapes

**DOI:** 10.1038/srep33100

**Published:** 2016-09-09

**Authors:** Jinbao Liao, Jiehong Chen, Zhixia Ying, David E. Hiebeler, Ivan Nijs

**Affiliations:** 1 Ministry of Education’s Key Laboratory of Poyang Lake Wetland and Watershed Research, Jiangxi Normal University, Ziyang Road 99, 330022 Nanchang, China; 2 College of Life Science, Key Laboratory of Poyang Lake Environment and Resource Utilization, Ministry of Education, Nanchang University, Nanchang 330029, China; 3 Department of Mathematics and Statistics, University of Maine, 333 Neville Hall, Orono, ME 04469, USA; 4 Centre of Excellence Plant and Vegetation Ecology, University of Antwerp (Campus Drie Eiken), Universiteitsplein 1, 2610 Wilrijk, Belgium

## Abstract

Habitat destruction, a key determinant of species loss, can be characterized by two components, patch loss and patch fragmentation, where the former refers to the reduction in patch availability, and the latter to the division of the remaining patches. Classical metacommunity models have recently explored how food web dynamics respond to patch loss, but the effects of patch fragmentation have largely been overlooked. Here we develop an extended patch-dynamic model that tracks the patch occupancy of the various trophic links subject to colonization-extinction-predation dynamics by incorporating species dispersal with patch connectivity. We found that, in a simple food chain, species at higher trophic level become extinct sooner with increasing patch loss and fragmentation due to the constraint in resource availability, confirming the trophic rank hypothesis. Yet, effects of fragmentation on species occupancy are largely determined by patch loss, with maximal fragmentation effects occurring at intermediate patch loss. Compared to the spatially explicit simulations that we also performed, the current model with pair approximation generates similar community patterns especially in spatially clustered landscapes. Overall, our extended framework can be applied to model more complex food webs in fragmented landscapes, broadening the scope of existing metacommunity theory.

Habitat destruction is one of the most influential factors contributing to species extinction^1^,^2^,^3^. Understanding its impacts has therefore become a central issue in ecology and conservation^4^,^5^. According to Fahrig^6^,^7^, habitat destruction involves two processes: patch loss and patch fragmentation, where the former refers to the decline of total available patch and the latter to the spatial arrangement of the remaining patches. To date, much theoretical as well as empirical work has explored the separate effects of habitat loss and fragmentation on species persistence and diversity^8^,^9^,^10^, and significant advances have been made in our understanding of how species respond to them^6^,^7^,^11^,^12^. Among those studies, however, very few have incorporated species trophic interactions and their consequences. Thus, how populations respond to habitat destruction in trophically linked communities has been largely overlooked^13^,^14^,^15^,^16^,^17^,^18^.

Recently, both empirical^19^,^20^,^21^,^22^ and modelling studies^23^,^24^ have started investigating the effects of patch loss on food web dynamics, based on the classic metapopulation models that track the patch occupancy of individual species at the landscape scale^25^,^26^,^27^. Most of these studies concluded that patch loss can reduce population sizes and trophic links, ultimately leading to species loss. However, these models first calculated the presence of prey and predator separately, and then applied predation by simply multiplying with the respective densities of prey and predator, while ignoring the fact that the trophic interaction should depend on their encounter frequency^23^,^28^. In addition, species within a local food chain can be affected not only by the extinction of their direct resource but also by all extinctions below it in the food chain. Keeping track of the rate of local extinctions for each species in the metacommunity thus requires determining the frequency of patch overlap between a given species and each of the species below it in the aggregate food web^29^. As such, Pillai *et al*.^29^,^30^,^31^ developed a novel patch-dynamic framework that tracks the changing patch occupancies of the various trophic links rather than those of the individual species, unlike previous metacommunity models^23^,^24^,^27^,^28^,^32^. Consequently, each suitable patch is either empty or occupied by a specific set of species with trophic links. From a metacommunity perspective, complex food webs are comprised of numerous local food chains or sub-webs linked by species dispersal. This is confirmed by a growing number of observations on natural ecosystems, where complex food webs emerge only on a regional scale, whereas simple food chains are observed if specific locations (patches) are considered in isolation^30^,^33^. Thus, the modelling framework of Pillai *et al*.^29^ allows one to study complex trophic networks undergoing habitat loss.

Currently, the patch-dynamic model developed by Pillai *et al*.^29^, however, still ignores details of the spatial arrangement of patches (e.g., patch fragmentation), which have been proven empirically to affect species persistence^34^,^35^,^36^. Using a single population model, Liao *et al*.^9^,^10^ already showed that species with contrasting dispersal abilities respond differently to patch fragmentation, with shorter-range dispersers responding more negatively than longer-range dispersers. Yet, Pillai *et al*.^29^ assumed global dispersal (i.e., uniform in space) for all species regardless of their trophic levels, which is relatively restrictive as species at different trophic levels often display distinct dispersal traits. For example, higher trophic level species tend to exhibit longer-range dispersal in nature^37^,^38^. Thus, the role of patch fragmentation in mediating the relationships between food webs and species dispersal remains untested and vaguely understood. Furthermore, we are still far from constructing mathematical models that predict how patch loss and spatial fragmentation separately act and interact in modifying food web dynamics, particularly along the axis of species dispersal ranges at different trophic levels. To address this problem, we propose an extended food chain model that incorporates both landscape fragmentation (see illustration in Fig. 1) and species dispersal, based on the modelling framework of Pillai *et al*.^29^. With this model, we investigate how metacommunities with different dispersal ranges at different trophic levels respond to patch availability and spatial connectivity.

## Results

We first use the extended patch-dynamic model (EPDM) to test how patch availability and connectivity interact in modifying species persistence at steady state in both bi- and tri-trophic systems (Figs 2 and 3). Generally, both increases of patch availability and connectivity promote species persistence and thus system stability, though the effects of patch availability are stronger (Figs 2b,c and 3b–d). Due to the trophic cascading effect, species at higher trophic levels become extinct sooner with reducing patch availability and connectivity, in spite of their dispersal superiority (Figs 2a and 3a). Regardless of the generally superior influence of patch availability over patch connectivity, patch connectivity can modify the effect of patch availability on species occupancy, especially at intermediate patch loss. Here, low patch connectivity leads to species extinction, while high connectivity maintains high patch occupancy (Figs 2 and 3). Furthermore, an increase of patch connectivity increases patch occupancy more for lower trophic level species, though with less increment at higher connectivity (Figs 2 and 3).

Next we check whether the patch occupancy dynamics predicted by the EPDM are in close proximity to those predicted by spatially explicit cellular automaton (CA) simulation (Figs 4 and 5). Generally, both models consistently predict that increasing patch connectivity enhances species persistence, with less enhancement at higher connectivity. Again, the effects of patch connectivity on patch occupancy are largely determined by patch availability, with maximal connectivity effects occurring at intermediate patch availability (Figs 4a–d and 5a–f). In fact, such connectivity effects are highly correlated with a change of the average patch cluster size (see Fig. S1 in Supplementary A). In some cases, landscapes with low patch availability but high connectivity can maintain higher species occupancy than landscapes with high patch availability but low connectivity (e.g., compare patch occupancy at (*s*, *q*_*s/s*_) = (0.3, 0.9) and (*s*, *q*_*s/s*_) = (0.5, 0.025) in Figs 4a–d and 5a–f). However, EPDMs generally overestimate patch occupancy relative to CA simulations, with more biased estimates occurring at lower patch availability (Figs 4a–d and 5a–f). This indicates that EPDM can predict connectivity effects more effectively when patch availability remains high. Moreover, an increase of patch availability linearly promotes species persistence in both models (Figs 4e–h and 5g–l). Compared to the CA model, EPDM can predict the impacts of patch availability on species occupancy similarly when patch connectivity is high, but largely underestimates it in highly fragmented landscapes (Figs 4e–h and 5g–l). As a result, EPDM can be used to qualitatively model patch dynamics especially in highly clustered landscapes with low patch loss.

## Discussion

We extend the patch-dynamic model derived by Pillai *et al*.^29^ to simulate spatial food chain dynamics in fragmented landscapes by combining species trophic level and dispersal trait. This extended model allows one to explore the effects of patch connectivity (or patch fragmentation), independent of patch availability (or patch loss), on the persistence of species as well as on trophic links in a food chain. Although some experiments have already demonstrated the critical role of habitat patch clustering in regulating metacommunity stability^39^,^40^,^41^, many food web models (i.e., non-spatial patch model) based on classical metacommunity theory still ignore spatial patch arrangement and species dispersal limitation^21^,^23^,^24^,^29^,^42^,^43^. In contrast to these metacommunity models, the current method uses a pair approximation method to directly model patch connectivity and local dispersal, providing a very different approach to model trophic networks in space. In accordance with observations in nature, we take account of increasing dispersal scales with increasing trophic position, ranging from small-scale neighbour dispersal in basal species, over within-patch dispersal in intermediate consumers, to global dispersal in top predators. When compared to the spatially explicit CA simulations (Figs 4 and 5), estimates of patch occupancy with the EPDM using pair approximation may deviate especially in highly fragmented landscapes with low patch availability. However, despite the approximations, EPDM does yield qualitatively similar results and the same general community patterns as CA models. In addition, our deterministic formulation generates simulation results quickly relative to CA models, which require large amounts of computer space and time to achieve approximations subject to stochastic fluctuations and deviations. To qualitatively study spatial effects on food chain dynamics in fragmented landscapes, our current model might be an alternative to the fully realistic CA models, as it can bridge the gap between non-spatial patch models (i.e., randomly-structured patch model; see Figs 2 and 3 marked with gray dashed lines at *s* = *q*_*s/s*_) and spatially explicit CA simulations by approximating their essential aspects (e.g., local dispersal and spatial patch connectivity).

In a bi- or tri-trophic system undergoing patch loss, we show that the higher trophic-level species become extinct sooner than the lower trophic-level species due to the bottom-up constraint (Figs 2, 3, 4, 5), confirming the trophic rank hypothesis^24^,^44^,^45^. This means that the effects of habitat loss will be noticed earlier in the higher trophic positions of the food chain. Accordingly, we suggest that the decline of top predators (in spite of their dispersal superiority) can be considered as a critical indicator for habitat deterioration, because patch loss leads to low patch occupancy of prey species, thereby resulting in food shortage for predators (a “bottom-up” constraint on predator occupancy). Different from Kondoh^24^, we do not find the top-down control that can result in a rapid increase in the abundance of intermediate consumers with increasing patch loss because of the release in top-down control, at least in a simple food chain. This is due to the fact that in the EPDM, intermediate consumers can only colonize the suitable patches already occupied by resources; hence they will always have lower patch occupancy than basal species, being constrained “bottom-up” by resources. Valladares *et al*.^46^ observed that overall leafminer herbivory decreases in small habitats, despite the possible release from parasitism, which supports a bottom-up view of patch loss effects in a plant-herbivore-parasitoid system. Moreover, increasing patch availability promotes species occupancy more obviously in spatially clustered landscapes (Figs 4 and 5), since an increase of patch connectivity can benefit the prey species with distance-limited dispersal, thereby providing more resources for the predators. This indicates that metacommunity models based on randomly structured landscapes (marked with gray dashed lines at *s* = *q*_*s/s*_ in Figs 2 and 3) may predict lower patch occupancy and therefore higher extinction risk for species in landscapes of highly connected patches.

Most previous modelling studies of food web dynamics have only considered patch loss while ignoring spatial patch configuration^21^,^23^,^24^,^29^,^42^,^43^. Yet, our EPDM suggests a significant impact of patch fragmentation on species persistence, with higher fragmentation resulting in higher species extinction risk and thus shorter food chain length (Figs 2 and 3). This result further supports the “bottom-up” prediction of shorter food chain length in less connected habitat patches, as confirmed by numerous observations^11^,^47^,^48^. For instance, Terborgh *et al*.^49^ found that vertebrate predators were lost from an experimental system of isolated islands in a flooded hydroelectric reservoir. Likewise, increasing isolation of fragments has been shown to reduce the food chain length of plant-herbivore-parasitoid systems^50^,^51^. Thus, our modelling result indicates that spatial effects of patch fragmentation *per se* may be another major factor driving biodiversity loss, in contrast to the conclusion of Yaacobi *et al*.^52^. Although all observations referred to above show the pattern predicted by bottom-up constraints on species persistence, none of these studies invoked this as an explanation^48^. Instead, the decline in food chain length due to patch fragmentation predicted by EPDMs should be attributed to a combination of greater extinction risk of predator than prey and different dispersal scales among trophic levels. In single population models, however, species with global dispersal are unaffected by patch fragmentation^9^,^10^. As a result, the observed decrease in the patch occupancy of the top predator with globally uniform dispersal must be ascribed to the negative fragmentation effect on its locally dispersing prey species (i.e., via the trophic cascading effect^53^). For instance, analyzing the plant-herbivore-parasitoid system revealed that the loss of specialist parasitoids results from a bottom-up cascade of species loss through plant and herbivore consumer levels^22^,^54^. Essentially, increasing patch fragmentation not only directly reduces the population growth of distance-limited dispersers, but also disrupts the trophic interactions between resource and consumer by blocking their dispersal between patch clusters, which thus further influences top predators via a trophic cascade.

The negative effects of patch fragmentation on food chain dynamics are largely determined by patch loss, with most pronounced fragmentation effects occurring at intermediate patch loss (Figs 4 and 5). In landscapes with low patch loss, the remaining habitat patches are always clustered together to form a small number of large patch clusters (see Fig. S1 in Supplementary A), thereby leading to relatively weak fragmentation effects. In landscapes with high patch loss, average patch cluster size only slightly increases with promoting patch connectivity, thus determining the weak connectivity effects on patch occupancy (Fig. S1). In some cases, species occupancy may reach higher levels in landscapes with low patch availability but high connectivity than in landscapes with high patch availability but low connectivity. This result implies that favorable patch configurations are able to compensate for overall patch loss and mitigate extinction risks, as empirically confirmed by Zabel and Tscharntke^55^. The fact that positive patch connectivity effects on species persistence gradually weaken in landscapes with high patch availability, brings an important ecological implication: in some cases, increasing patch connectivity as much as possible may not be the optimal strategy for maintaining species diversity in trophically linked metacommunities if conservation cost is included.

Our current model only simulates a simple food chain, while most metacommunities in nature are more complex. They consist of dozens of species and may contain other types of predation (e.g., omnivory and intraguild predation) as well as other types of interactions such as competition, mutualism (e.g., pollination) and facilitation. As a consequence, some modelling predictions may be inapplicable because of the monotonicity of the trophic structure in a simple food chain. For instance, an omnivorous top predator in a multi-trophic system facing patch loss and fragmentation might not always go extinct sooner than intermediate consumers because of its feeding on different trophic levels, contradicting the trophic rank hypothesis^24^,^44^,^45^. Furthermore, increasing patch connectivity might not always be an optimal strategy for maximizing species diversity in a food web including exploitative competition, owing to a dispersal-competition tradeoff mediated by patch fragmentation^30^. Besides, using a Lotka-Volterra model, Pimm and Lawton^56^ predicted that the stability of simple food chains will decline with increasing number of trophic levels probably because of the potential scaling effects (e.g., compare Figs 2 and 3). Finally, for model simplicity, we only considered three ideal types of dispersal scaling determined by trophic level (i.e., neighbour dispersal, dispersal within patch clusters and global dispersal), which is relatively restrictive as species in nature display a broad range of movement behaviors^57^. However, these dispersal modes have long been used in ecological models and are considered meaningful to analyze dispersal effects on eco-dynamics^8^,^9^,^10^,^58^, as they can generate approximately the same results as more realistic dispersal modes.

In conclusion, we present an extended patch-dynamic framework for food chain dynamics by incorporating species dispersal ranges along the axis of trophic level, in order to explore how species respond to patch availability and connectivity both separately as well as to their interaction. Both patch availability and connectivity play a critical role in determining food chain length, following the principle of bottom-up constraint. This indicates that basal species, even when they are highly distance-limited in their dispersal, can tolerate much more patch loss and fragmentation relative to high trophic level species with dispersal superiority. Furthermore, the effects of patch connectivity largely depend on patch availability, with maximal connectivity effects occurring at intermediate patch loss. Future study can extend this theoretical framework to more complex food web metacommunities, for example, food web modules with omnivory, apparent competition or intraguild predation. Overall, the development of extended patch-dynamic models for food webs in fragmented landscapes provides a significant step towards broadening the scope of existing metacommunity theory.

## Methods

### Fragmented landscape generation

Similar to Hiebeler^8^,^58^, we simulate fragmented landscapes with a two-dimensional square lattice of size *L* × *L* = 100 × 100 cells (*L* is the length of the lattice), which acts like a torus to avoid the edge effect. Each cell representing one patch can be either empty or occupied by multiple populations with trophic links, i.e., by a specific set of trophically-linked species. To introduce landscape fragmentation, we define two types of habitat: suitable (*s*) and unsuitable (*u*), where only *s-*patches (proportion *s*, i.e., patch availability) can accommodate species, whilst *u*-patches (proportion *u*, i.e., patch loss) are unsuitable for any species establishment (*s* + *u* = 1). From the large number of patch connectivity indices that have been formulated to describe spatial patch patterns^8^,^59^,^60^,^61^, we select the local density to characterize the connectivity for a given patch type^8^,^61^. For *s*-patches, for example, the patch connectivity equals *q*_s/s_ = *ρ*_*ss*_/*s*, with *ρ*_*ss*_ the pair density denoting the probability that a randomly chosen pair of nearest neighbours are both *s*-patches. Here we adopt the von Neumann neighbourhood approach where each patch has four orthogonally adjacent patches (*z* = 4). Consequently, the local density *q*_*s/s*_ is the conditional probability that the neighbour of an *s*-patch is also an *s*-patch. The degree of fragmentation for *s*-patches is negatively related to their connectivity, defined as 1-*q*_*s/s*_. According to Hiebeler^8^,^58^, the allowable range of patch connectivity for *s*-patches decreases with increasing patch availability (*s*), with


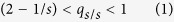


**Figure 1:**
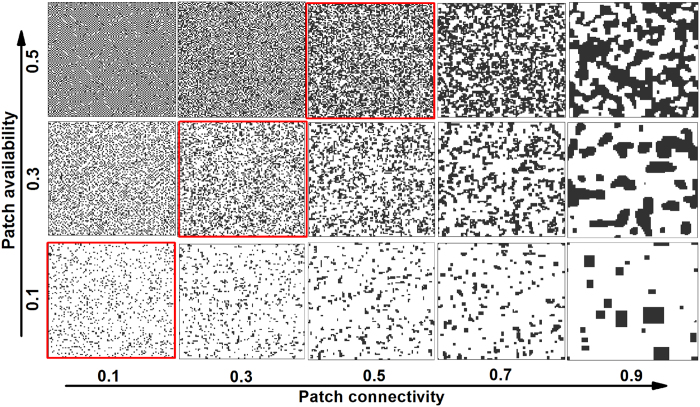
Artificial fragmented landscapes consisting of two patch types (black – suitable, white – unsuitable) in a square lattice of size *L* × *L* = 100 × 100 cells with each cell representing one patch, differentiated by varying both patch availability (*s*) and connectivity (*q*_*s/s*_). Each image shows a typical configuration for the given properties. In the special case with *q*_*s/s*_ = *s*, both patch types are randomly distributed (marked with red square), while the cases of *q*_*s/s*_ > *s* and *q*_*s/s*_ < *s* respectively represent spatially clustered and over-dispersed patterns of suitable patches.

**Figure 2:**
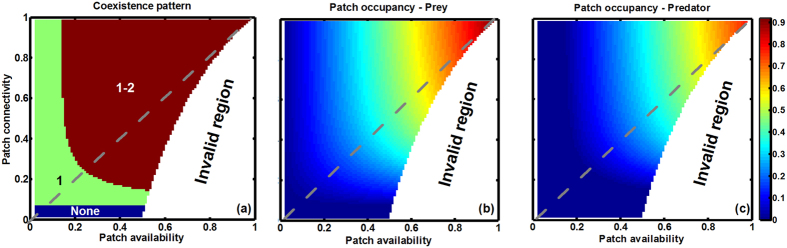
Interactive effects of patch availability (*s*) and connectivity (*q*_*s/s*_) on species persistence at steady state in a prey-predator system, modelled with EPDM. (**a**) Species coexistence pattern (1 – prey, 2 – predator, None – extinction of all species, and gray dashed line with *s* = *q*_*s/s*_ – randomly structured landscapes), (**b**) global patch occupancy of the prey species, and (**c**) global patch occupancy of the predator. Note that the range of patch connectivity shrinks with increasing patch availability, yielding the invalid region (see equation (1)). Parameter values: species colonization rate *c*_1_ = *c*_2_ = 1, intrinsic extinction rate *e*_1_ = *e*_2_ = 0.05 and top-down extinction rate due to predation *μ*_21_ = 0.025.

**Figure 3:**
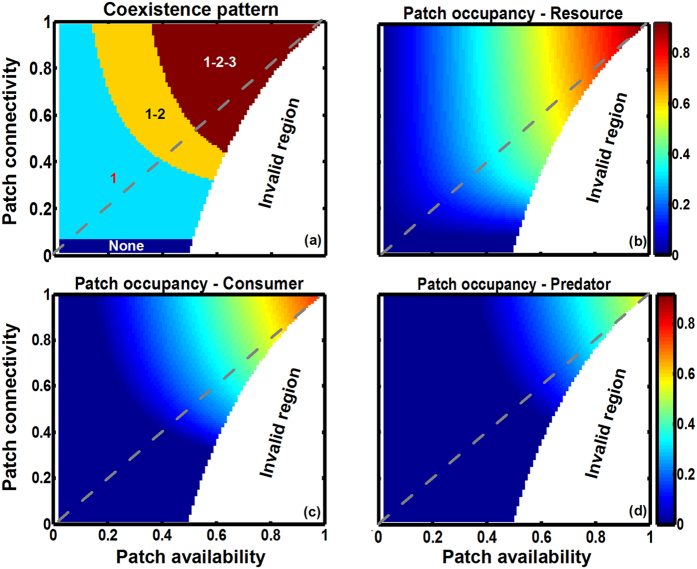
Interactive effects of patch availability (*s*) and connectivity (*q*_*s/s*_) on species persistence in a simple food chain (1 → 2 → 3), modelled by EPDM. (**a**) Species coexistence pattern (1 – Resource, 2 – Consumer, 3 – Predator, None – extinction of all species), and (**b–d**) global patch occupancy respectively of resource, consumer and predator. Gray dashed lines with *s* = *q*_*s/s*_ denote randomly structured landscapes. Parameter values (see Fig. 2 for definitions): *c*_1_ = *c*_2_ = *c*_3_ = 1, *e*_1_ = *e*_2_ = *e*_3_ = 0.05 and *μ*_32_ = *μ*_21_ = 0.025. Invalid region: see equation (1).

**Figure 4:**
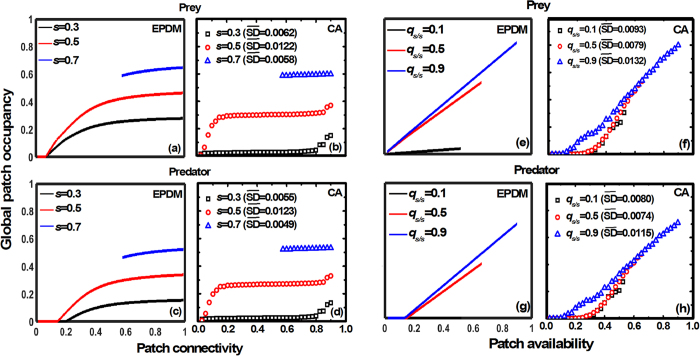
Individual effects of patch availability (*s*) and connectivity (*q*_*s/s*_) on the steady-state patch occupancy of a prey-predator system, simulated by both EPDM and CA models (average of 100 replicates; SD (standard deviation) of replicates are omitted for figure clarity; 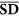 denotes the mean of all SDs in each case). Left panels (a–d): patch occupancy of both prey and predator by varying patch connectivity at fixed patch availability (*s* = 0.3, 0.5, 0.7). Right panels (e–h): patch occupancy of both prey and predator by varying patch availability at fixed connectivity (*q*_*s/s*_ = 0.1, 0.5, 0.9). Other parameter values: see Fig. 2. Note that the range of patch connectivity shrinks with increasing patch availability (see equation (1)).

**Figure 5:**
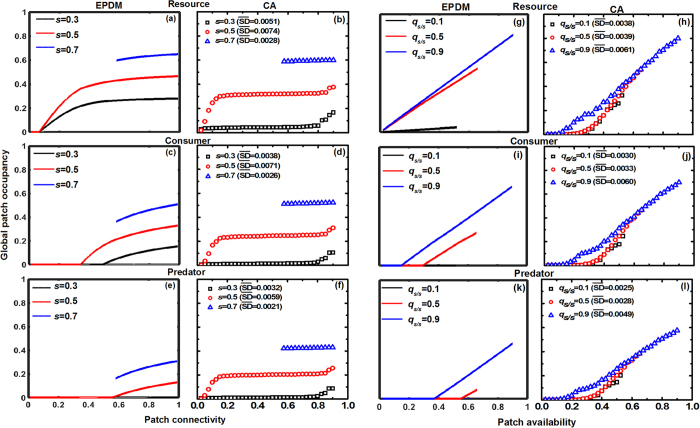
Individual effects of patch availability (*s*) and connectivity (*q*_*s/s*_) on patch occupancy at steady state in a simple food chain (Resource 1 → Consumer 2 → Predator 3), simulated by both EPDM and CA models (average of 100 replicates; SD (standard deviation) of replicates are omitted for figure clarity; 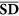
denotes the mean of all SDs in each case). Left panels (a–f): species occupancy by varying patch connectivity *q*_*s/s*_ at fixed patch availability *s* (=0.3, 0.5, 0.7). Right panels (g–l): species occupancy by varying *s* at fixed *q*_*s/s*_ (=0.1, 0.5, 0.9). Other parameter values: see Fig. 3. Again, the range of patch connectivity shrinks with increasing patch availability (see equation (1)).

Based on the orthogonal neighbouring correlation algorithm^8^,^58^, we can generate a diversity of fragmented landscapes by independently varying patch availability *s* or/and patch connectivity *q*_s/s_ (Fig. 1). This provides a convenient framework to investigate the individual effects of these landscape characteristics on food web dynamics. In the special case of *s* = *q*_s/s_, the two patch types *s* and *u* are randomly distributed across the whole landscape, while the cases of *s* < *q*_s/s_ and *s* > *q*_s/s_respectively represent clustered and over-dispersed patterns of the two patches.

### An extended patch-dynamic model (EPDM) for a prey-predator system

We first consider a simple prey-predator system with species 1 being eaten by species 2 (denoted by 1 → 2) as an example to introduce the extended patch-dynamic model. In this case, an occupied patch can have two possible food chain configurations: 1 or 1 → 2, as the predatory species 2 cannot survive without the prey species 1 in a local patch. Since species at higher trophic levels typically display longer-range dispersal^37^,^38^,^62^, we assume that the prey species has local dispersal and can only colonize the neighbouring empty suitable patches (using von Neumann neighbourhood with *z* = 4), while the predatory species has global dispersal and can randomly establish in all suitable patches that contain only the prey species but not itself. As a result, the dispersal of the prey species between patch clusters (defined as a number of suitable patches connected based on the principle of 4-nearest neighbours) is inhibited by unsuitable patches, while the spread of predators is not influenced^9^,^10^. Thus we can describe the patch-dynamics of: (i) the prey species with a pair approximation (PA) model, which has already proved qualitatively useful in characterizing neighbouring correlation in lattice-structured models^8^,^61^,^63^,^64^,^65^,^66^,^67^; and (ii) the predator species with a non-spatial mean-field approximation (MFA). Consequently, by modifying the non-spatial patch-dynamic model introduced by Pillai *et al*.^29^, we derive an extended patch-dynamic model for a prey-predator system in fragmented landscapes as


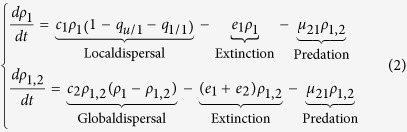


in which all parameters are defined as in Table 1.

The patch-dynamics of the prey species in equation (2) include three elements: prey population colonization (*c*_1_) with neighbouring dispersal, intrinsic population extinction (*e*_1_) and increased extinction caused by predation (*μ*_21_). The factor (1−*q*_u/1_−*q*_1/1_) = *q*_s/1_ denotes the conditional probability that a suitable unoccupied *s*-patch neighbours a randomly chosen 1-patch (i.e., a suitable patch occupied by species 1) across the entire landscape, as only three possible neighbouring states exist for a target 1-patch: 1 (including 1 → 2), *u* and *s*. Similarly, the patch occupancy dynamics of the trophic link 1 → 2 in equation (2) consist of three parts: the growth of the trophic link 1 → 2 via global dispersal of predators (*c*_2_), the loss of the 1 → 2 links in patches due to the intrinsic mortality of either prey (*e*_1_) or predator (*e*_2_), and the top-down extinction of species 1 due to predation by species 2 (with a rate *μ*_21_). The factor (*ρ*_1_−*ρ*_1,2_) is the proportion of 1-patches occupied only by prey species but not predators, indicating that predators can only colonize these 1-patches.

Since the terms *q*_1/1_ and *q*_*u*/1_ change over time due to the colonization-extinction-predation dynamics of the prey species, we further describe their transition rates in equations (B7-B8) (see Supplementary B). Thus, equations (2) and (B7-B8) construct a closed prey-predator system incorporating patch availability and connectivity.

### An extended patch-dynamic model (EPDM) for a simple food chain

Similar to the above prey-predator system, we derive the dynamics of a simple food chain (basal species 1 →  intermediate consumer 2 →  top predator 3) as


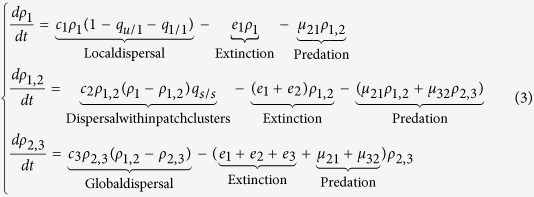


In this tri-trophic system (see Table 1 for parameters), an occupied patch can have three possible food chain configurations: 1, 1 → 2 and 1 → 2 → 3, as the intermediate consumer cannot survive in a local patch without the resource, and the top predator cannot locally survive without prey species. In equation (3), the dynamics of the resource include three elements: colonization via neighbour dispersal, intrinsic population extinction and predation by intermediate consumers. The intermediate consumers (equation (3)) are assumed to randomly disperse only within patch clusters, i.e., their spread between patch clusters is restricted by unsuitable patches. Thus the growth of 1–2 links is multiplied with patch connectivity *q*_*s/s*_ to approximately indicate the restriction of dispersal between patch clusters. The dispersal limiting factor *q*_*s/s*_, equal to the mean crowding index^63^,^68^, characterizes the average clustering degree of suitable patches, indicating both mean patch cluster size and configurational fragmentation. For example, if *q*_*s/s*_ ≈1, i.e., all suitable patches are clustered together to form one large patch cluster, then the intermediate consumers have dispersal opportunities similar to those of the top predators with global dispersal (see equation (3)). If *q*_*s/s*_ ≈ 0, i.e., suitable patches are highly fragmented, the dispersal of consumers is extremely limited. The loss of 1–2 links in equation (3) consists of two aspects: intrinsic extinction of species 1 or 2, and predation between 1 → 2 links or 2 → 3 links. Concerning top predators, we assume they can globally disperse to any suitable patches with only prey species (i.e., *ρ*_1, 2_−*ρ*_2, 3_), thus unsuitable patches cannot block their dispersal between patch clusters. Similarly, the loss of the trophic link 2 → 3 (i.e., of 1 → 2 → 3) in equation (3) includes: intrinsic extinction of species 1, 2 or 3, as well as top-down extinction due to over-predation between 1 → 2 links or 2 → 3 links. This is because in a local patch with 1 → 2 → 3 links, if resource species 1 dies, then consumer species 2 cannot survive, and top predator 3 also would become extinct immediately though it has no direct trophic link with basal species 1. Note that we have *ρ*_2_ = *ρ*_1, 2_ and *ρ*_3_ = *ρ*_2, 3_ in this tri-trophic system.

In order to form a closed mathematical system for the simple food chain, we need to further derive the dynamics of *q*_1/1_ and *q*_*u*/1_. These are approximately the same as equations (B7) and (B8), because when considering the loss of the pair 1–1 patches caused by predation (with *μ*_21_*ρ*_1, 2_/*ρ*_1_ in equation (B3); see Supplementary B), we approximately assume that species 2 is randomly distributed on all the 1-patches across the entire landscape. Therefore, equations (3) and (B7-B8) completely describe the patch-dynamics for a tri-trophic system in fragmented landscapes.

With the model, we use numerical methods (via ODE45 in MATLAB) to derive the non-trivial solutions for symmetrical parameter combinations, that is, all species have the same parameter values (see more details in the source code in Supplementary C). In each simulation, all species in the food chain are initialized at low patch occupancy, with lower occupancy at higher trophic level. Each case is run for enough time to achieve a steady state (using *ρ*_*i*_ < 0.0001 as species extinction threshold; *i* = 1, 2 or 3). Using the symmetrical parameter combinations that we choose for all subsequent simulations, we find that 10000 time steps are enough in most cases to reach system stability. Throughout this paper, we explore a broad range of biologically realistic parameter combinations and the results presented for these combinations are qualitatively robust. By varying patch availability (0 < *s* < 1) and connectivity (0 < *q*_*s/s*_ < 1), we seek to explore how patch loss and fragmentation separately affect food chain persistence.

### Cellular automaton (CA) simulations

We also use a spatially explicit cellular automaton simulation (CA) to model the patch-dynamics of a simple food chain, in order to check whether both CA and EPDM models can produce similar community patterns in fragmented landscapes. As a discrete-time patch occupancy model, the CA simulation is implemented on a square lattice of size *L* × *L* = 100 × 100 cells (i.e., patches) which acts like a torus to avoid the edge effect, and fragmented landscapes characterized by both patch availability (*s*) and connectivity (*q*_s/s_) are generated by varying the orthogonal neighbour correlation between suitable and unsuitable patches^8^,^58^ (see above). The initial population distribution of the basal species within the landscape is determined randomly with low patch occupancy, ignoring populations landing on unsuitable patches. Then intermediate consumers or/and top predators randomly establish in the suitable patches already occupied by their prey species with a lower patch occupancy. As intermediate consumers can only disperse at random within patch clusters, we first use the ‘bwconncomp’ package in MATLAB to recognize each patch cluster in a given fragmented landscape based on the principle of 4-nearest neighbours. During each time step, three independent events occur for each species (see source code in Supplementary D):

- Species colonization. For example, a target empty *s*-patch can be occupied by basal species (via neighbour dispersal) with a probability 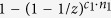, where *n*_1_ is the number of neighbouring patches occupied by basal species (0 ≤ *n*_1_ ≤ 4) and *z* = 4. Similarly, a target patch with basal species can be occupied by intermediate consumers (via random dispersal within patch clusters) with a probability of 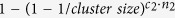, in which *n*_2_ is the number of patches occupied by consumers within a given patch cluster. Likewise, a target patch with 1 → 2 link can be colonized by predatory species 3 (via global dispersal) with a probability 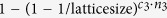, where *n*_3_ is the number of patches occupied by top predators across the landscape.
- Intrinsic extinction. Each species population in a local patch undergoes intrinsic extinction with a probability *e*_*i*_ (i = 1, 2 or 3).
- Top-down extinction. In each trophic link, prey species also undergo predation by their predators, resulting in the top-down extinction risk for prey with a rate *μ*_*ji*_ (species *i* eaten by species *j*; *i* → *j*).

Note that if a prey species goes extinct in a local patch, the predator within that patch also becomes extinct immediately. Each simulation is run until all species achieve a steady state (100000 time steps are enough for system stability at *e*_*i*_ = 0.05), and each case is simulated with 100 replicates in order to reduce effects of stochastic fluctuations. In each replicate, the fragmented landscape is regenerated with the same properties but keeping *s* and *q*_s/s_ constant, and the mean patch occupancy for each species at steady state is calculated by averaging on the final 10000 time steps. The average of the 100 replicates ultimately yields an estimate of global patch occupancy for both species and trophic links at steady state.

## Additional Information

**How to cite this article**: Liao, J. *et al*. An extended patch-dynamic framework for food chains in fragmented landscapes. *Sci. Rep.* **6**, 33100; doi: 10.1038/srep33100 (2016).

## Supplementary Material

Supplementary Information

## Figures and Tables

**Table 1:** Model parameters.

Parameter	Interpretation
*u*	Fraction of unsuitable patches
*s*	Fraction of suitable patches
*ρ*_*i*_	Global patch occupancy of species *i*
*ρ*_*i, j*_	Fraction of the suitable patches occupied by both species *i* and *j* with trophic interactions, or patch occupancy of the trophic link *i* → *j*, with “,” indicating the trophic link within a local patch
*ρ*_*kl*_	Probability in a randomly chosen pair of neighbouring patches that one patch is *k* and the other is *l* (pair density; *k*, *l∈ *{*s*, *u*, 1, 2, 3})
*q*_*k/l*_	Conditional probability that the neighbour of a *l*-patch is a *k*-patch (local density; *k*, *l∈ *{*s*, *u*, 1, 2, 3})
*q*_*s/s*_	Clustering degree of suitable patches before species introduction (i.e., patch connectivity), indirectly indicating mean patch cluster size and configurational fragmentation
*c*_*i*_	Species colonization rate
*e*_*i*_	Species extinction rate
*μ*_*ji*_	The top-down extinction rate of species *i* eaten by species *j*
