# Corrigendum: An extended patch-dynamic framework for food chains in fragmented landscapes

**DOI:** 10.1038/srep35329

**Published:** 2016-10-19

**Authors:** Jinbao Liao, Jiehong Chen, Zhixia Ying, David E. Hiebeler, Ivan Nijs

Scientific Reports 6: Article number: 3310010.1038/srep33100; published online: 09 09 2016; updated: 10 19 2016

The Acknowledgements section in this Article is incomplete.

“This study is supported by the Opening Fund of Key Laboratory of Poyang Lake Wetland and Watershed Research (Jiangxi Normal University), Ministry of Education (No. PK2015004 and TK2016002)”.

should read:

“This study was funded by the DFG through grant FOR 1748 and the Ministry of Science and Culture of Lower Saxony in the project “Biodiversity-Ecosystem Functioning across marine and terrestrial ecosystems”. This study is also supported by the Opening Fund of Key Laboratory of Poyang Lake Wetland and Watershed Research (Jiangxi Normal University), Ministry of Education (No. PK2015004 and TK2016002). We also thank Prof. Dr. Bernd Blasius and Dr. Daniel Bearup for helpful discussions”.

