# Supplementary material for: An extended patch-dynamic framework for food chains in fragmented landscapes
Source: Sci Rep. 2016 Sep 9;6:33100. doi: 10.1038/srep33100 (PMC5016810; doi:10.1038/srep33100)
Supplement: Supplementary Information [file srep33100-s1.pdf]

An extended patch-dynamic framework for food chains in fragmented landscapes

Jinbao Liao[1,*], Jiehong Chen[1], Zhixia Ying[2], David E. Hiebeler[3] & Ivan Nijs[4]

[1]Ministry of Education's Key Laboratory of Poyang Lake Wetland and Watershed Research, Jiangxi Normal University, Ziyang Road 99, 330022 Nanchang, China

[2]College of Life Science, Key Laboratory of Poyang Lake Environment and Resource Utilization, Ministry of Education, Nanchang University, Nanchang 330029, China

[3]Department of Mathematics and Statistics, University of Maine, 333 Neville Hall, Orono, ME 04469, USA

[4]Centre of Excellence Plant and Vegetation Ecology, University of Antwerp (Campus Drie Eiken), Universiteitsplein 1, 2610 Wilrijk, Belgium

[*]**Corresponding author:** Dr. Jinbao Liao (jinbaoliao@163.com)

Tel.: +86-(0)791-88133622 Fax: +86-(0)791-88120538

Supplementary A

Effects of patch availability and connectivity on average patch cluster size

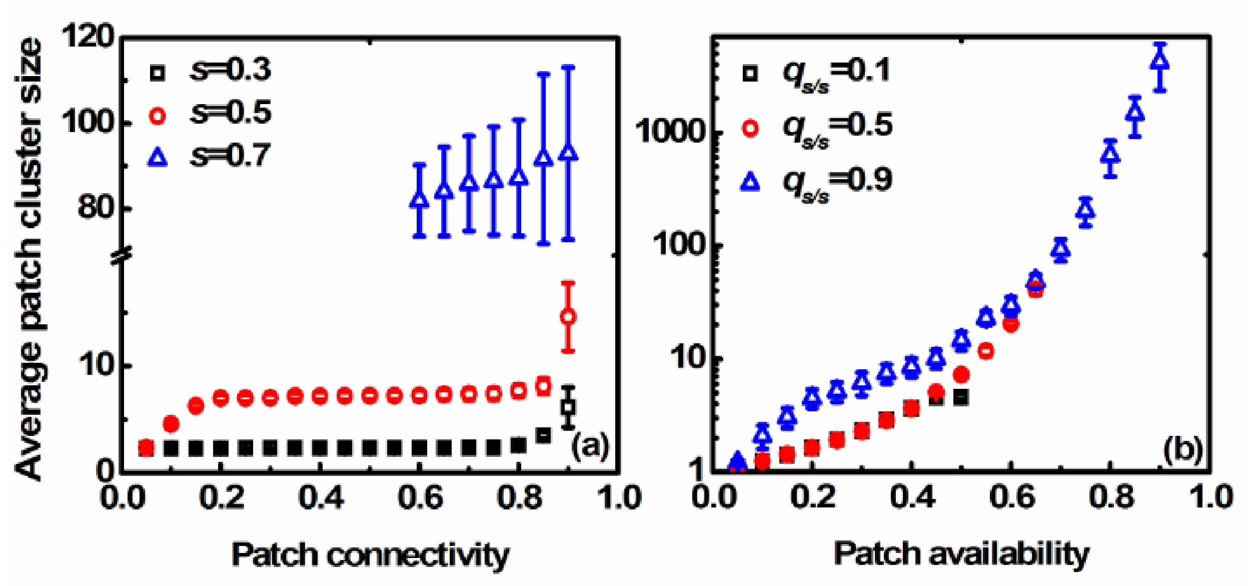

Figure S1. Effects of patch availability (*s*) and connectivity ($q_{s/s}$) on average patch cluster size (average ± SD of 100 replicates) in fragmented landscapes with a square lattice of size $L \times L = 100 \times 100$ cells. In each replicate, the landscape is regenerated with the same properties (i.e., keeping *s* and $q_{s/s}$ constant). Left panel (a): average patch cluster size by varying $q_{s/s}$ at fixed *s* (=0.3, 0.5, 0.7); and right panel (b): average patch cluster size by varying *s* at fixed $q_{s/s}$ (=0.1, 0.5, 0.9). Again, the range of patch connectivity shrinks with increasing patch availability (see equation (1)).

Supplementary B

Deriving the dynamics of $q_{1/1}$ ***and*** $q_{u/1}$

According to the definition of local density, we have $q_{1/1} = \rho_{11} / \rho_1$ and $q_{u/1} = \rho_{u1} / \rho_1$. By differentiation, we thus obtain

$$\frac{dq_{1/1}}{dt} = \frac{1}{\rho_1}\frac{d\rho_{11}}{dt} - \frac{q_{1/1}}{\rho_1}\frac{d\rho_1}{dt} \qquad \text{(B1)}$$

and

$$\frac{dq_{u/1}}{dt} = \frac{1}{\rho_1}\frac{d\rho_{u1}}{dt} - \frac{q_{u/1}}{\rho_1}\frac{d\rho_1}{dt}. \qquad \text{(B2)}$$

To completely construct the dynamics of $q_{1/1}$, we first derive the dynamics of ρ_{11} as

$$\frac{d\rho_{11}}{dt} = 2c_1\rho_{s1}\left(\frac{1}{z} + \frac{z-1}{z}q_{1/s1}\right) - 2\rho_{11}\left(e_1 + \mu_{21}\rho_{1,2} / \rho_1\right), \qquad \text{(B3)}$$

where the first term is the state transition from the pair *s*-1 (or 1-*s*) to 1-1 via local dispersal (*s*-empty suitable patches, and 1-suitable patches occupied by species 1), in which the pairs *s*-1 and 1-*s* are distinguished though their pair densities are equal. This change can be achieved by one of the two pathways: the colonization of the *s*-patch from the 1-patch in an *s*-1 pair or the colonization of this *s*-patch from one of its (*z*-1) other neighbours. The latter pathway should be multiplied by $q_{1/s1}$, which is the conditional probability that another 1-patch neighbours the *s*-patch in a *s*-1 pair (using the von Neumann neighbourhood with *z*=4). The second term represents the loss of the pairs 1-1→1-*s* (or *s*-1), due to intrinsic extinction or top-down

predation. As predator species 2 with global dispersal can randomly establish only in the suitable patches with prey species 1, we can directly adopt the ratio $\rho_{1,2}/\rho_1$ to represent the proportion of prey species that may go extinct due to the top-down predation.

Similarly, we can construct the dynamics of ρ_{1u} as follows

$$\frac{d\rho_{1u}}{dt} = c_1\rho_{su}\frac{z-1}{z}q_{1/su} - \rho_{1u}\left(e_1 + \mu_{21}\rho_{1,2}/\rho_1\right), \tag{B4}$$

in which the first term on the right side denotes the transition rate of *s*-*u*→1-*u* by colonizing the empty *s*-patch in a *s*-*u* pair from one of its z-1 other neighbours, and the factor $q_{1/su}$ is the conditional probability that a 1-patch is the neighbour of a *s*-patch in the *s*-*u* pair. The second term is the loss of the pairs 1-*u*→*s*-*u* because of intrinsic extinction or top-down predation.

In view of the pair approximation principle, we obtain the following constraints

$$\begin{cases} q_{i/jk} \approx q_{i/j} \\ q_{s/1} = 1 - q_{u/1} - q_{1/1} \\ q_{1/s} = \rho_1\left(1 - q_{u/1} - q_{1/1}\right)/(1 - u - \rho_1) \\ \rho_{su} = uq_{s/u} = u(1 - q_{u/u} - \rho_1 q_{u/1}/u) \end{cases}, \tag{B5}$$

In a fragmented landscape, we have

$$\begin{cases} u + s = 1 \\ q_{u/u} = 1 - q_{s/u} = \dfrac{1 - 2s + sq_{s/s}}{1 - s} \end{cases}, \tag{B6}$$

where patch availability *s* and patch connectivity $q_{s/s}$ here are constant for a given landscape, including the already occupied *s*-patches.

Combining equations (B1-B6) with (2a), we thus obtain the dynamics of $q_{1/1}$ and $q_{u/1}$ as

$$\begin{cases} \dfrac{dq_{1/1}}{dt} = c_1\left(1 - q_{1/1} - q_{u/1}\right)\left(\dfrac{1}{2} + \dfrac{3}{2}\rho_1 \dfrac{1 - q_{u/1} - q_{1/1}}{s - \rho_1} - q_{1/1}\right) - q_{1/1}\left(e_1 + \mu_{21} \cdot \rho_{1,2} / \rho_1\right) & \text{(B7)} \\ \dfrac{dq_{u/1}}{dt} = c_1\left(\dfrac{3}{4} \cdot \dfrac{s - sq_{s/s} - \rho_1 q_{u/1}}{s - \rho_1} - q_{u/1}\right) \cdot \left(1 - q_{u/1} - q_{1/1}\right) & \text{(B8)} \end{cases}$$

Supplementary C

Source code - EPDM model for a simple food chain in fragmented landscapes

```
function y=islandsystem(t,x)
global c1 c2 c3 pe1 qe1e1 e1 e2 e3 u21 u32
y=zeros(5,1);
y(1)=c1*x(1)*(1-x(4)-x(5))-e1*x(1)-u21*x(2);
y(2)=c2*x(2)*(x(1)-x(2))*qe1e1-e2*x(2)-e1*x(2)-u21*x(2)-u32*x(3);
y(3)=c3*x(3)*(x(2)-x(3))-(e1+e2+e3+u21+u32)*x(3);
y(4)=c1*(1-x(4)-x(5))*(0.5+1.5*x(1)*(1-x(4)-x(5))/(pe1-x(1))-x(4))-x(4)*(e1+u21*x(2)/x(1));
y(5)=(0.75*c1*(1-pe1)*((1-x(1)*x(5)/(1-pe1)-(1-2*pe1+pe1*qe1e1)/(1-pe1))/(pe1-x(1)))-c1*x(5))*(1-x(4)-x(5));
-----------------------------------------------------------------------------------
clear
global c1 c2 c3 pe1 qe1e1 e1 e2 e3 u21 u32
c1=1; c2=1; c3=1;%species colonization rate
e1=0.05; e2=0.05; e3=0.05;%species intrinsic mortality rate
u21=0.025; u32=0.025;%top-down extinction rate
tn=[0,10000]; y0=[0.01 0.005 0.0025 0.08 0.0005]; %Initial parameter values
axispe1=0.01:0.01:0.99; axisqe1e1=0.01:0.01:0.99; % range of patch availability and connectivity

[X,Y]=meshgrid(axispe1,axisqe1e1);%pe1-patch availability and qe1e1-patch connectivity
[nn,mm]=size(X);%nn – connectivity of unsuitable patches and mm - patch loss
   RDensity=zeros(nn,mm)-1; %resource
   CDensity=zeros(nn,mm)-1;%consumer
   PDensity=zeros(nn,mm)-1;%predator
   RRDensity=zeros(nn,mm)-1;%local density of resource species
   REDensity=zeros(nn,mm)-1;%conditional probability qu/1
   Periodicarea=zeros(nn,mm)-1;% test system oscillation or whether system reaches stability

  for n1=1:nn
      for    n2=1:mm
            pe1=axispe1(n2);qe1e1=axisqe1e1(n1);
            if (pe1<0.5)||(pe1>=0.5&&qe1e1>2-1/pe1) % constraint for patch availability and connectivity
[t,y]=ode45('islandsystem',tn,y0); %algorithm of EPDM equations
if max(y(end-100:end,1))>mean(y(end-100:end,1))+0.0005
                        Periodicarea(n1,n2)=1;
                  else
                        Periodicarea(n1,n2)=0;
end

RDensity(n1,n2)=mean(y(end-100:end,1));
RRDensity(n1,n2)=mean(y(end-100:end,4));
REDensity(n1,n2)=mean(y(end-100:end,5));
CDensity(n1,n2)=mean(y(end-100:end,2));
PDensity(n1,n2)=mean(y(end-100:end,3));
else
RDensity(n1,n2)=NaN;
RRDensity(n1,n2)=NaN;
```

```
REDensity(n1,n2)=NaN;
CDensity(n1,n2)=NaN;
PDensity(n1,n2)=NaN;
            end
        end
    end

    RDensityi=find(RDensity<0.0001);%extinction threshold of resource
    RDensity(RDensityi)=0;
    CDensityi=find(CDensity<0.0001);%extinction threshold of consumer
    CDensity(CDensityi)=0;
    PDensityi=find(PDensity<0.0001);%extinction threshold of predator
    PDensity(PDensityi)=0;

figure,surf(X,Y,Periodicarea),view(0,90),colorbar
xlabel('pe1'),ylabel('qe1e1')
figure,surf(X,Y,RDensity),view(0,90),colorbar
xlabel('pe1'),ylabel('qe1e1')
figure,surf(X,Y,CDensity),view(0,90),colorbar
xlabel('pe1'),ylabel('qe1e1')
figure,surf(X,Y,PDensity),view(0,90),colorbar
xlabel('pe1'),ylabel('qe1e1')

Z=RDensity;
ii0=find(RDensity<0.0001);%resource does not exist
ii1=find((RDensity>0.0001)&(CDensity<0.0001));%resource exists but consumer goes extinct
ii2=find((RDensity>0.0001)&(CDensity>0.0001)&(PDensity<0.0001));%resource & consumer coexist
but predator goes extinct
ii3=find((RDensity>0.0001)&(CDensity>0.0001)&(PDensity>0.0001));%All species coexistence
Z(ii0)=0; Z(ii1)=1; Z(ii2)=2; Z(ii3)=3;
figure,surf(X,Y,Z),view(0,90),colorbar
xlabel('pe1'),ylabel('qe1e1')
```

Supplementary D

Source code - CA simulation for a simple food chain in fragmented landscapes

```
function [Predator,Consume,resource,pp,pc,pr]=stept(A,Predator,Consume,resource)
c1=1; c2=1; c3=1; %species colonization rate
e1=0.025; e2=0.025; e3=0.025; %species intrinsic mortality rate
u21=0.05; u32=0.05; %Top-down extinction rate

%patch cluster reorganization
[n1,n2]=size(A);
output = bwconncomp(A,4); %based on 4-nearest neighbourship
CC=output. PixelIdxList; % patch locations in the landscape
Npatches=output.NumObjects;%patch numbers

%cacluate the global density of Predator, Consumer, Resource
    pp=0;pc=0;pr=0;pe2=0;
     for ii=1:n1
         for jj=1:n2
              if Predator(ii,jj)==2
                 pp=pp+1;
              end
              if Consume(ii,jj)==2
                 pc=pc+1;
              end
              if resource(ii,jj)==2
                 pr=pr+1;
              end
              if A(ii,jj)==0
                 pe2=pe2+1;
              end
         end
     end
      pp=pp/(n1*n2);pc=pc/(n1*n2);
      pr=pr/(n1*n2); pe2=pe2/(n1*n2);

  Consume2=Consume;
  resource2=resource;
  Predator2=Predator;

  % %%%%%Top Predator
  % state transition from 2 (predator) to 1(unoccupied by predator)
  % and transition from 1(unoccupied by predator) to 2(predator)
  for ii=1:n1
        for jj=1:n2
             if Predator2(ii,jj)==2
          p1=rand(1); p2=rand(1); p3=rand(1); p4=rand(1); p5=rand(1);
          if (p1<e1)|(p2<e2)|(p3<e3)|(p4<u21)|(p5<u32) %intrinsic mortality and top-down extinction
             Predator(ii,jj)=1;
          end
```

```
                    end
                    if (Predator2(ii,jj)==1)&(Consume2(ii,jj)==2)&(resource2(ii,jj)==2)
                         p1=rand(1);
                          if p1<1-(1-0.0001)^(c3*pp*10000) %patch occupancy
                              Predator(ii,jj)=2;
                          end
                    end
           end
     end

%%%%%Intermediate consumer
% state transition from 2 (consumer) to 1(unoccupied by consumer)
  for ii=1:n1
           for jj=1:n2
                   if Consume2(ii,jj)==2
                   p1=rand(1); p2=rand(1); p3=rand(1); p4=rand(1);
                   if    (p1<e2)|(p2<e1)|(p3<u21)|(Predator2(ii,jj)==2 & p4<u32)    %
                       Consume(ii,jj)=1;
                   end
                   end
           end
  end

% transition from 1(unoccupied by consumer) to 2(consumer)
      for ii= 1:Npatches % Npatches is the patch cluster number
      xylabel=CC{ii}; % the ii-th patch cluster location in the 2-D matrix or the 1-D matrix
      [nvalue,nvalue2]=size(xylabel); % nvalue is the patch cluster size of patch cluster i
      region_C=sum(Consume2(xylabel)-1);% region_C is the consumer number in the ii-th patch cluster
      region_R=sum(resource2(xylabel)-1);% region_R is the resource number in the ii-th patch cluster
      region_Rdensity=region_R/nvalue;% region_Rdensity is the local density in the ii-th patch cluster
      region_Cdensity=region_C/nvalue;
              for jj=1:nvalue
                   jj1=xylabel(jj);
                   if Consume2(jj1)==1 & resource2(jj1)==2
                       p1=rand(1);
                       if p1<1-(1-1/nvalue)^(c2*region_C)
                       Consume(jj1)=2;
                       end
                   end
              end
      end

%%%% %Resource
% transition from 1(unoccupied by resource) to 2(resource)
for ii=1:n1
    for jj=1:n2
      if ii==1&&jj==1
      x=[resource2(100,jj),resource2(ii+1,jj),resource2(ii,100),resource2(ii,jj+1)];
      end
      if ii==1&&jj==100
      x=[resource2(100,jj),resource2(ii+1,jj),resource2(ii,jj-1),resource2(ii,1)];
```

```
     end
     if ii==100&&jj==100
     x=[resource2(ii-1,jj),resource2(1,jj),resource2(ii,jj-1),resource2(ii,1)];
     end
     if ii==100&&jj==1
     x=[resource2(ii-1,jj),resource2(1,jj),resource2(ii,100),resource2(ii,jj+1)];
     end
     if ii==1&&(jj>1&&jj<100)
     x=[resource2(100,jj),resource2(ii+1,jj),resource2(ii,jj-1),resource2(ii,jj+1)];
     end
     if ii==100&&(jj>1&&jj<100)
     x=[resource2(ii-1,jj),resource2(1,jj),resource2(ii,jj-1),resource2(ii,jj+1)];
     end
     if jj==100&&(ii>1&&ii<100)
     x=[resource2(ii-1,jj),resource2(ii+1,jj),resource2(ii,jj-1),resource2(ii,1)];
     end
     if jj==1&&(ii>1&&ii<100)
     x=[resource2(ii-1,jj),resource2(ii+1,jj),resource2(ii,100),resource2(ii,jj+1)];
     end
     if ii>1&&ii<100&&jj>1&&jj<100
     x=[resource2(ii-1,jj),resource2(ii+1,jj),resource2(ii,jj-1),resource2(ii,jj+1)];
     end
     x1=0;
       for kk=1:4
         if x(kk)==2
         x1=x1+1;% the number of occupied sites
         end
       end
     if resource2(ii,jj)==1
     p1=rand(1);
     if p1< 1-(1-1/4)^(x1*c1)
     resource(ii,jj)=2;
     end
     end
    end
end

% transition from 2(resource) to 1(unoccupied by resource)
for ii=1:n1
  for jj=1:n2
    if resource2(ii,jj)==2
    p1=rand(1);p2=rand(1);
      if (p1<e1)|(Consume2(ii,jj)==2&p2<u21)
      resource(ii,jj)=1;
      end
    end
  end
end

for ii=1:n1
 for jj=1:n2
```

```
if resource(ii,jj)==1
Consume(ii,jj)=1;
Predator(ii,jj)=1;
end
if Consume(ii,jj)==1
Predator(ii,jj)=1;
end
end
end
```

```
a=load('data(0.5,0.5).txt');% Fragmented landscape by taking s=0.5 and qs/s=0.5 for example
%see landscape generation algorithm in Hiebeler 2000 (Ecology) and 2007 (J. Math. Biol.)
n1=100;n2=100;%lattice size
A= reshape(a,n1,n2);%
% The initial value of resource, consume, predator
resource=A;%resource value 0,1,2 respectively expresses non-habitat, suitable habitat but without resource, and suitable site with resource
Consume=A;% Consume value 0,1,2 respectively denotes nonhabitat, suitable habitat but without consumer, and suitable site with consumer
Predator=A;% Predator value 0,1,2respectively denotes nonhabitat, suitable habitat but without predator, and suitable site with predator
P_initial_resource=0.5;%initial resource occupancy
P_initial_Consume=0.5;%initial consumer occupancy
P_initial_Predator=0.5;%initial predator occupancy
    for ii=1:n1
        for jj=1:n2
            if A(ii,jj)==1
                p1=rand(1); p2=rand(1); p3=rand(1);
                if P_initial_resource>p1
                resource(ii,jj)=2;
                end
                if (P_initial_Consume>p2)&(resource(ii,jj)==2)
                Consume(ii,jj)=2;
                end
                if (P_initial_Predator>p3)&(Consume(ii,jj)==2)
                Predator(ii,jj)=2;
                end
            end
        end
    end

nn=100000;%time steps
Pdensity=zeros(1,nn);Cdensity=zeros(1,nn);rdensity=zeros(1,nn);
% predator, consumer, resource over time

for ii=1:nn
[Predator,Consume,resource,pp,pc,pr]=stept(A,Predator,Consume,resource);
Pdensity(ii)= pp; Cdensity(ii)=pc;rdensity(ii)=pr;
end

%average over the final 10000 time steps
```

```
mean(rdensity(90000:100000))
mean(Cdensity(90000:100000))
mean(Pdensity(90000:100000))
```
